# Altered Localization of Hybrid Incompatibility Proteins in *Drosophila*

**DOI:** 10.1093/molbev/msz105

**Published:** 2019-04-30

**Authors:** Jacob Carter Cooper, Andrea Lukacs, Shelley Reich, Tamas Schauer, Axel Imhof, Nitin Phadnis

**Affiliations:** 1 School of Biological Sciences, University of Utah, Salt Lake City, UT; 2 Faculty of Medicine, Institute for Molecular Biology, Biomedical Center (BMC), LMU Munich, Germany; 3 Center for Integrated Protein Science Munich (CIPSM), Ludwig-Maximilians-Universität Müchen, Munich, Germany

**Keywords:** speciation, hybrid incompatibilities, telomeres

## Abstract

Understanding the molecular basis of hybrid incompatibilities is a fundamental pursuit in evolutionary genetics. In crosses between *Drosophila melanogaster* females and *Drosophila simulans* males, an interaction between at least three genes is necessary for hybrid male lethality: *Hmr ^mel^*, *Lhr ^sim^*, and *gfzf ^sim^*. Although HMR and LHR physically bind each other and function together in a single complex, the connection between *gfzf* and either of these proteins remains mysterious. Here, we show that GFZF localizes to many regions of the genome in both *D. melanogaster* and *D. simulans*, including at telomeric retrotransposon repeats. We find that GFZF localization at telomeres is significantly different between these two species, reflecting the rapid evolution of telomeric retrotransposon copy number composition between the two species. Next, we show that GFZF and HMR normally do not colocalize in *D. melanogaster*. In interspecies hybrids, however, HMR shows extensive mis-localization to GFZF sites, thus uncovering a new molecular interaction between these hybrid incompatibility factors. We find that spreading of HMR to GFZF sites requires *gfzf ^sim^* but not *Lhr ^sim^*, suggesting distinct roles for these factors in the hybrid incompatibility. Finally, we find that overexpression of HMR and LHR within species is sufficient to mis-localize HMR to GFZF binding sites, indicating that HMR has a natural low affinity for GFZF sites. Together, these studies provide the first insights into the different properties of *gfzf* between *D. melanogaster* and *D. simulans*, and uncover a molecular interaction between *gfzf* and *Hmr* in the form of altered protein localization.

## Introduction

The formation of new species from a single ancestral population involves the evolution of reproductive isolating barriers (Dobzhansky 1937; Coyne and Orr 2004). Intrinsic postzygotic reproductive barriers, such as the sterility or inviability of hybrids, are caused by deleterious genetic interactions known as hybrid incompatibilities. Understanding the evolutionary forces that generate such barriers between species requires understanding the properties of incompatibility genes—namely, what are their native functions within species, and how do they interact with each other? Despite decades of studies, a system of species with detailed molecular understanding of their hybrid incompatibilities remains elusive.

**Fig. 1.**
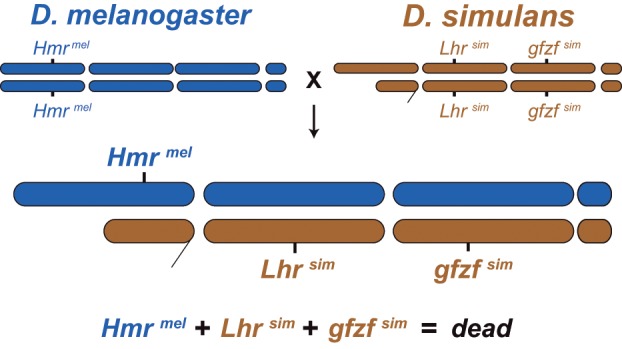
Three genes are required for F1 male lethality. *Hmr^mel^, Lhr^sim^*, and *gfzf^sim^* are all required for hybrid F1 male lethality between *Drosophila melanogaster* and *Drosophila simulans*.

One of the longest studied interspecies hybridizations is between the fruit fly *Drosophila melanogaster* and its closest sister species, *Drosophila simulans*. When *D. melanogaster* females are crossed to *D. simulans* males, the hybrid F1 males die during larval development (Sturtevant 1919). These F1 hybrid males lack imaginal disc tissues, and do not undergo sufficient growth to trigger pupation; hybrid F1 females live to adulthood but are sterile (Sánchez and Dübendorfer 1983). Three genes are essential for the inviability of these hybrid F1 males: *Hybrid male rescue* from *D. melanogaster* (*Hmr*^ *mel*^) (Hutter and Ashburner 1987; Barbash et al. 2003), *Lethal hybrid rescue* from *D. simulans* (*Lhr*^*sim*^) (Watanabe 1979; Brideau et al. 2006), and GST-containing FLYWCH zinc finger protein from *D. simulans* (*gfzf*^*sim*^) (Phadnis et al. 2015) (fig. 1). These three alleles interact genetically to form a single hybrid incompatibility, and all three incompatible alleles must be simultaneously present for hybrid F1 male lethality. Loss-of-function or reduced expression of any individual incompatible allele is sufficient to rescue the viability of hybrid F1 males. *Hmr*^ *mel*^ is lethal to hybrid males whereas *Hmr*^ *sim*^ does not cause hybrid male inviability. Conversely, *Lhr* ^ *sim*^ and *gfzf*^* sim*^ are incompatible alleles that cause hybrid F1 male lethality, whereas the corresponding alleles of *Lhr*^ *mel*^ and *gfzf*^ *mel*^ do not contribute to hybrid F1 male lethality. The *D. melanogaster*–*D. simulans* hybridization thus represents one of the best understood systems in terms of the identities of the genes involved in hybrid lethality (Barbash 2010).

*Hmr* and *Lhr* (also known as heterochromatin protein 3 [HP3]) are not essential for viability within species (Watanabe 1979; Hutter and Ashburner 1987). Biochemical studies have shown that HMR and LHR physically bind to each other and function together in a complex with HP1a (Brideau et al. 2006; Thomae et al. 2013; Satyaki et al. 2014). Null mutants of *Hmr* and *Lhr* in *D. melanogaster* show defects in sister chromatid detachment during anaphase (Blum et al. 2017) and show derepression of transcripts from several families of transposable elements and satellite DNA (Thomae et al. 2013; Satyaki et al. 2014). Both proteins are overexpressed in hybrid F1 males, and hybrids display a pattern of derepression of transcripts from transposable elements and satellite DNA (Thomae et al. 2013; Satyaki et al. 2014). The molecular mechanism for how *Hmr* and *Lhr* interact with *gfzf* to form a lethal hybrid incompatibility remains unclear.

*gfzf* encodes a protein that contains four FLYWCH zinc finger domains, and a GST domain (Dai et al. 2004). In contrast to HMR and LHR, there is no evidence for a physical interaction of either protein with GFZF. Unlike *Hmr* and *Lhr*, *gfzf* is essential for viability in *D. melanogaster* (Provost et al. 2006). Loss-of-function mutants of *gfzf* die shortly after hatching into larvae, in a pattern reminiscent of hybrid F1 male lethality (Provost et al. 2006; Bolkan et al. 2007). *gfzf* has been identified repeatedly in several genetic screens, including those designed to identify suppressors of the *Killer-of-prune* system (Provost et al. 2006), cell-cycle regulation (Ambrus et al. 2009), DNA damage induced cell-cycle checkpoints (Kondo and Perrimon 2011), Ras/MAPK signaling (Ashton-Beaucage et al. 2014), and Polycomb complex regulation (Gonzalez et al. 2014). One potential explanation for the role of *gfzf* in such a variety of processes comes from a recent study where *gfzf* was identified as a transcriptional coactivator through an association with Motif1 binding protein, and shown to bind the transcriptional start sites of genes relevant to the genetic screens where *gfzf* was identified as a hit (Baumann et al. 2018).

Here, we investigate the properties and functional differences of *gfzf* between *D. melanogaster* and *D. simulans*, and in F1 hybrids between these species. First, our cytological analyses show that there is little difference in protein localization of GFZF between the two species except at telomeric sequences. This differential binding of GFZF at telomeres persists in interspecies hybrids. These differences in localization appear to be due to changes in sequence composition at telomeres between *D. melanogaster* and *D. simulans*, and not due to species-specific amino acid changes in GFZF. Second, we investigate the pattern of GFZF and HMR colocalization, and find that the two proteins do not normally colocalize in *D. melanogaster*. In interspecies hybrids, however, HMR shows extensive mis-localization to GFZF sites, and this mis-localization requires the presence of *gfzf*^ *sim*^. Third, we find by ChIP-Seq that overexpression of HMR and LHR causes HMR to mis-localize to GFZF binding sites, indicating that HMR has some natural low affinity for GFZF sites. Together, these results provide the first hints of the allele-specific properties of *gfzf*, and the novel molecular interactions between *Hmr* and *gfzf* in hybrids between *D. melanogaster* and *D. simulans*.

## Results

### Different Localization Patterns of GFZF at *D. melanogaster* and *D. simulans* Telomeres

**Fig. 2.**
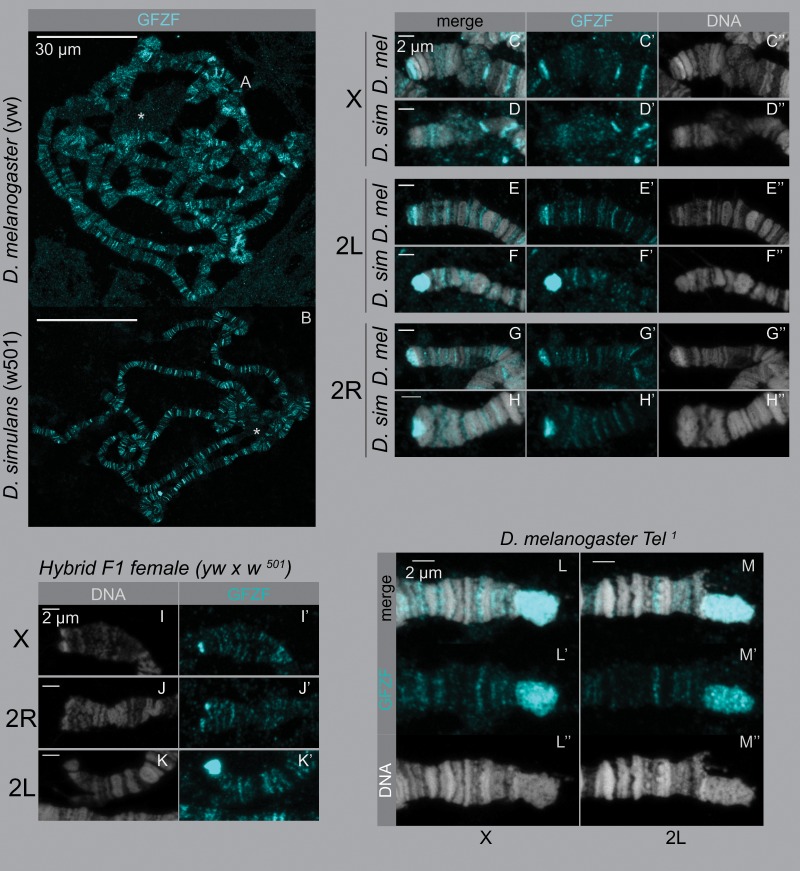
GFZF localization is different at chromosome ends in *Drosophila melanogaster* and *Drosophila simulans*. Polytene chromosomes were stained with anti-GFZF and Hoechst. (*A*–*B*) Whole polytenes from *D. melanogaster* and *D. simulans*. In both species, GFZF localizes to multiple bands across the genome. The star on each polytene indicates the chromocenter. (*C*-*H*) Magnified ends of polytene chromosomes from *D. melanogaster* and *D. simulans*, stained for GFZF and DNA. (*I*–*K*) Hybrid polytene chromosome ends of X, 2R, and 2L. There is a differential localization pattern on X and 2L, but not on 2R. (*H*–*I*) GFZF binds telomere retrotransposon repeats. The *Tel^1^* mutant has extended telomeres due to additional replication of the telomeric retrotransposons. In F1 progeny between *yw* and *Tel^1^*, the GFZF signal extends with the elongated *Tel^1^* telomere.

Though *gfzf* is a chromatin associated factor that has many binding sites in the genome (Baumann et al. 2018), it is not clear if this property is consistent between *D. melanogaster* and *D. simulans*. To determine whether *gfzf*^ *mel*^ and *gfzf*^ *sim*^ show differences in chromatin localization patterns, we developed an antibody that can recognize the GFZF protein from both species (supplementary fig. S1, Supplementary Material online). We performed immunostaining for natively expressed GFZF across multiple tissues, and found that it consistently localizes to the nucleus in *D. melanogaster* and *D. simulans* (supplementary figs. S2 and S3, Supplementary Material online). As we did not observe differences in the nuclear localization of GFZF at the cellular level, we next investigated the patterns of GFZF localization on polytene chromosomes. Consistent with previous reports, we found that GFZF localizes to many discrete bands on polytene chromosomes in *D. melanogaster* (fig. 2*A* and *B*). However, we also noticed strong localization of GFZF to the ends of several chromosomes, most notably the ends of the X and 2R chromosomes in *D. melanogaster*. These patterns are clearly visible both in our images and also in a re-examination of previously reported polytene analyses (Baumann et al. 2018).

HMR localizes to the telomere capping complex along with other locations in the genome (Thomae et al. 2013; Satyaki et al. 2014). To test whether GFZF localizes to the telomere capping complex or the retrotransposon repeats that comprise the telomeres of *Drosophila*, we used a *Tel*^*1*^ mutant strain that has extended telomeric retrotransposon repeats (Siriaco et al. 2002). An increase of GFZF staining in *Tel*^*1*^ polytenes and the lack of colocalization with HP1 indicate that GFZF localizes to the retrotransposon repeats at telomeres but not to the telomere cap (fig. 2*L* and *M*, supplementary fig. S4, Supplementary Material online).

We further investigated the patterns of GFZF localization on polytene chromosomes in *D. simulans* and found that most bands of GFZF binding appear qualitatively similar between the two species. However, we uncovered striking species-specific differences in GFZF localization at telomeres (fig. 2*C*–H). On the X chromosome, there is a bright band of staining on the end in *D. melanogaster* but not in *D. simulans*. This pattern is reversed at the end of 2L, where there is a bright patch of staining in *D. simulans* but not *D. melanogaster*. The end of chromosome arm 2R shows similar staining in both species, whereas the ends of 3L and 3R have little staining in either species. These patterns of species-specific GFZF staining at the ends of chromosomes seen in pure *D. melanogaster* and *D. simulans* individuals also persists in F1 hybrids between the two species (fig. 2*I*–*K*, supplementary fig. S5, Supplementary Material online).

**Fig. 3.**
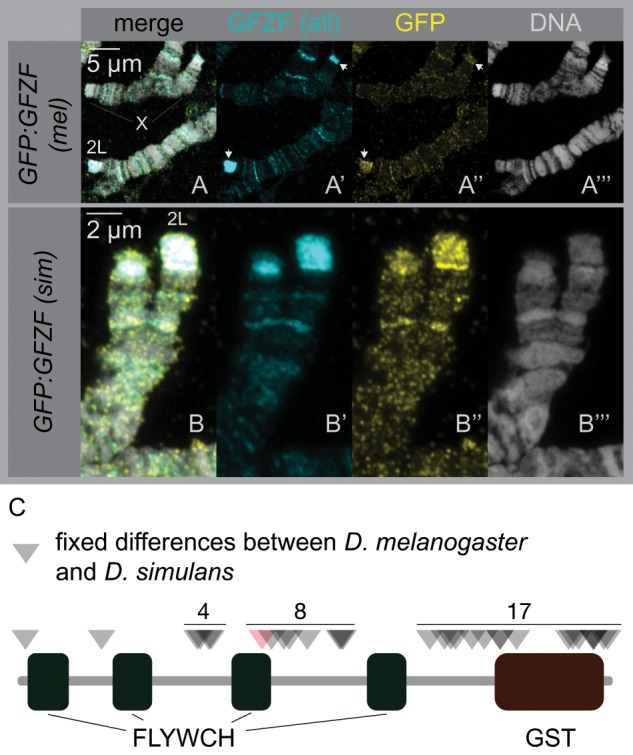
GFZF does not have species-specific binding patterns in polytenes. (*A*–*B*) F1 hybrid polytene chromosomes are stained for total GFZF and an EGFP–GFZF transgene from each individual species. In both cases, the anti-GFZF and anti-GFP signals colocalize, showing that there is not a difference in GFZF binding due to protein sequence. (*A*) *Drosophila melanogaster* GFP: GFZF(mel) crossed to *Drosophila simulans w^501^*, X chromosome (top) with chromosome 2L (bottom). (*B*) *D. melanogaster w^1118^* crossed to *D. simulans* GFP: GFZF(sim), images are of chromosome 2L. (*C*) Diagram of the fixed amino acid differences in *D. melanogaster* and *D. simulans*. FLYWCH domains and the GST domain are annotated. Triangles represent the position of the fixed differences, and the single red triangle is the only fixed difference in a FLYWCH domain.

In F1 hybrids, GFZF localization on the *D. melanogaster* polytene chromosomes versus the *D. simulans* polytene chromosomes is dramatically different at telomeres, but otherwise appears identical at other regions of the genome. There is precedence for hybrid incompatibility proteins differentially binding DNA in hybrids, such as in the case of *Odysseus* which causes hybrid sterility in introgression males between *D. simulans* and *Drosophilamauritiana* (Bayes and Malik 2009). Both *gfzf*^ *mel*^ and *gfzf*^ *sim*^ are expressed in F1 hybrids, raising the possibility that they may drive ectopic localization of one-another (Phadnis et al. 2015). Given the protein coding differences in GFZF between the two species, a simple hypothesis is that the two alleles of GFZF may show preferential binding to telomeres from its native species. To determine whether these patterns of differential localization reflect differences in binding specificities of the two alleles of GFZF or sequences composition differences at telomeres between the two species, we performed immunofluorescence studies using transgenes that carry GFP-tagged GFZF from either species. We found that both alleles of GFZF localize to telomeres in hybrids, with little or no allele-specific differences in their localization patterns (fig. 3*A* and *B*). This overlap in localization is supported by examining the fixed coding differences between *gfzf*^ *mel*^ and *gfzf ^sim^*(fig. 3*C*). Only one of the 31 fixed coding differences between the two alleles falls in any of the FLYWCH domains, suggesting that the nucleic acid binding properties may not be different between the two forms. These results suggest that the differential binding patterns of GFZF at telomeres are not caused by differences in the binding specificities of the two forms of GFZF, and instead reflect the rapid divergence of telomeric sequence composition between species (Danilevskaya et al. 1998; Anderson et al. 2008).

### Altered Localization of HMR in F1 Hybrids Requires GFZF^s^^im^

**Fig. 4.**
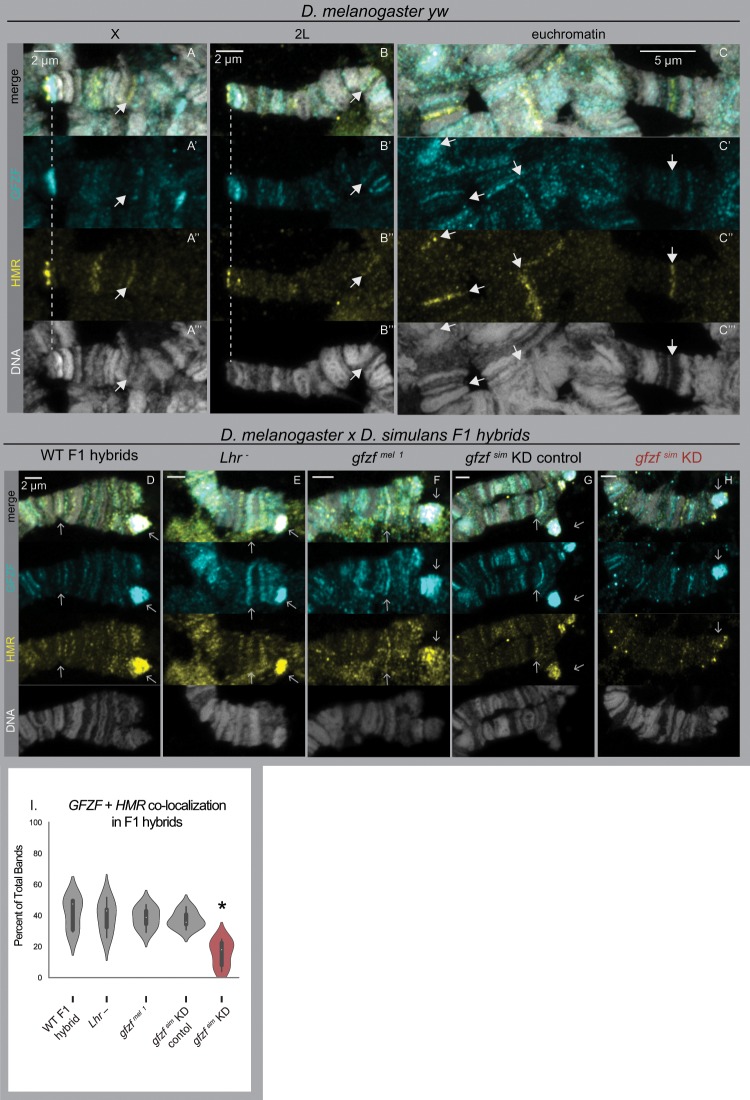
Aberrant colocalization of GFZF and HMR in F1 hybrids requires GFZF^sim^. (*A*–*C*) *Drosophila melanogaster* (*yw* strain) polytene chromosomes are stained with anti-GFZF and anti-HMR. There are few instances of colocalization, even when they are in close proximity at the telomeres. (*D*–*H*) Hybrid F1 female polytene chromosomes, stained for anti-GFZF and anti-HMR. All images show the telomeres of 2L. White arrows indicate the tip of the chromosome and a band of significant colocalization for (*D*–*G*). (*D*) WT hybrids of *yw* and *w^501^*. HMR spreads out to GFZF bands. (*E*) Hybrids of *yw* crossed to *Lhr^1^*. (*F*) Hybrids of *gfzf^1^* crossed to *w^501^*. (G) Hybrid of *gfzf^1^* crossed to *w^501^*. (*G*–*H*) Hybrids from UAS-RNAi(*gfzf^sim^*); Actin5C-GAL4/CyO crossed to *w^501^*. GAL4 versus CyO samples were identified by inversions on chromosome 2. In GAL4 expressing samples, HMR spreading is reduced and colocalization has decreased. (*I*) Quantification of colocalization. The number of HMR/GFZF co-occurring bands is presented as a percentage of the total bands recorded. *P < 0.00039 by a permutation test between given sample and the WT control, all other samples did not significantly differ from WT. *n* = 6 for each condition.

Our immunofluorescence studies with polytene chromosomes indicate that GFZF and HMR are in close proximity to each other at telomeres, where they localize to the telomeric retrotransposon repeats and the telomere capping complex respectively. To directly examine interactions between GFZF and HMR, we costained polytene chromosomes with antibodies for each protein. In *D. melanogaster*, we found that GFZF and HMR form separate bands through most of the genome, and rarely localize to the same band (fig. 4*A*–*C*). At telomeres, it appears that HMR localizes immediately distal to GFZF, consistent with the pattern that we observed with GFZF and HP1a. We attempted to detect the native localization of LHR in these contexts. Although an LHR antibody exists that has been used previously for biochemical assays (Thomae et al. 2013), we failed to detect an LHR immunofluorescent signal in our experiments.

Previous observations show that HMR spreads to many additional sites in the polytene chromosomes of F1 hybrids (Thomae et al. 2013). We costained hybrid polytenes for GFZF and HMR and found that many of these new HMR chromatin sites colocalize with GFZF sites (fig. 4*D*). In euchromatic regions of F1 hybrids, there are more instances of HMR banding, where HMR appears to spread to many more locations throughout the genome. At the telomeres, HMR is no longer confined to the telomere cap, and instead leaks into the GFZF regions. Not all GFZF sites costain for HMR, suggesting that this overlap of HMR and GFZF localization in F1 hybrids is incomplete.

To test if the altered localization of HMR to GFZF sites in F1 hybrids is dependent on GFZF, we depleted allele-specific versions of GFZF and measured HMR and GFZF colocalization. We used an Actin5C-GAL4 driver to knockdown *gfzf*^ *sim*^ expression via RNAi, which has previously been shown to be sufficient to rescue hybrid male viability (Phadnis et al. 2015). Interestingly, reducing the expression of *gfzf*^ *sim*^ greatly reduced the spreading of HMR into euchromatic regions of the genome, causing the HMR banding pattern to largely revert to the pattern seen in pure species individuals (fig. 4*H*, supplementary fig. S6, Supplementary Material online). We next tested whether a null allele of *Lhr*^ *sim*^, which is also sufficient to rescue hybrid male viability, similarly restored normal HMR localization. Here, we examined polytene chromosomes of hybrids from a cross between *D. melanogaster yw* females to *D. simulans Lhr^1^* males. In contrast to our results with reducing the expression of *gfzf*^ *sim*^, we find that the loss-of-function mutation in *Lhr*^ *sim*^ does not prevent the spreading of HMR to the euchromatic regions of the genome (fig. 4*E*). Next, to test whether removing *gfzf*^ *sim*^ versus removing *gfzf*^ *mel*^ showed differences in their effect on HMR mis-localization, we crossed females carrying a null allele of *D. melanogaster gfzf* to *D. simulans* males, and found that removing GFZF^mel^ does not stop the spreading of HMR (fig. 4*F*). We quantified these results by counting instances of overlapping peaks along polytene chromosomes, and found that reduction of GFZF ^sim^ is the only perturbation that significantly decreases the altered localization of HMR and to GFZF sites (fig. 4*I*). These results indicate that the mis-localization of HMR is dependent on GFZF ^sim^, but not on GFZF^mel^ or LHR ^sim^.

### Overexpressed HMR in *D. melanogaster* Mis-Localizes to GFZF Sites

We next asked if experimental manipulation within species could recapitulate an ectopic interaction between GFZF and HMR. A simple explanation for the mis-localization of HMR to GFZF binding sites in hybrids is that HMR may have some natural affinity for GFZF sites, but does not normally localize to these sites when *Hmr* is expressed at a low level. HMR and LHR are known to have higher protein levels in hybrids than in their parent species (Thomae et al. 2013; Satyaki et al. 2014)—in this context, higher expression of HMR may be responsible for its localization to GFZF binding sites. Alternatively, overexpressed HMR may form new protein–protein interactions with GFZF and localize to GFZF binding sites.

**Fig. 5.**
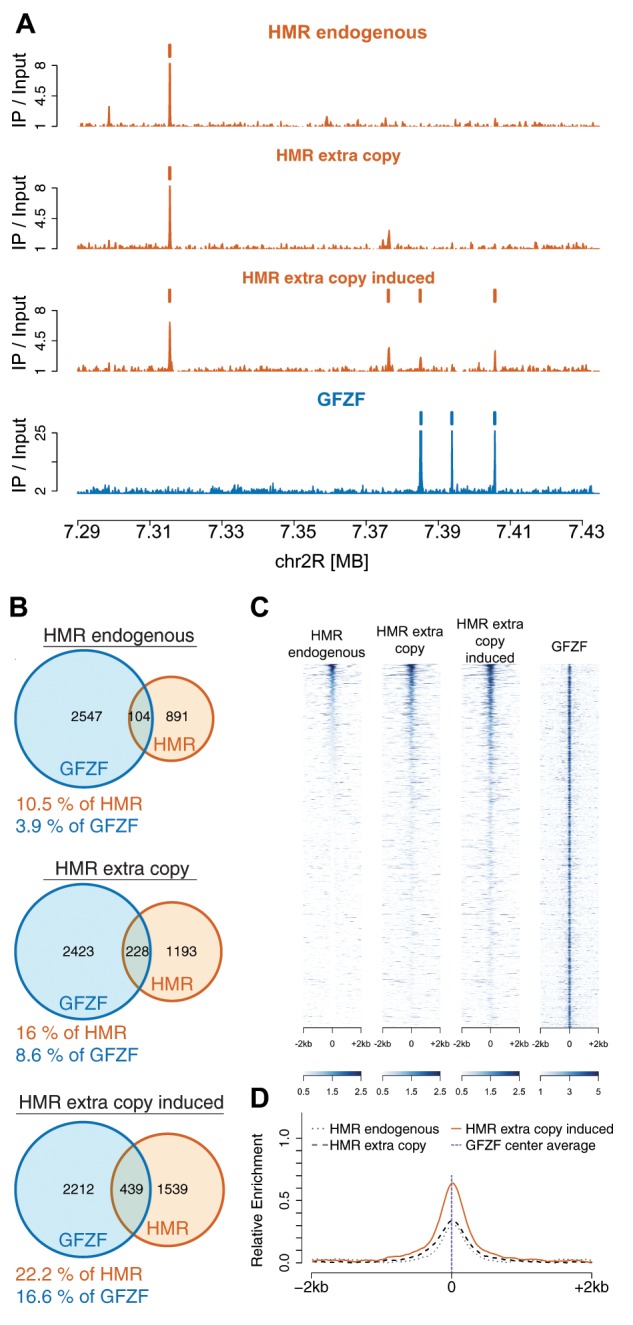
Overexpression of *Hmr* and *Lhr* in *Drosophila melanogaster* causes HMR binding to GFZF chromatin sites. (*A*) A representative example of HMR and GFZF ChIP-Seq plot over a region of chromosome 2R, focused on the *Dscam1* locus. This snap-shot presents an example of the different patterns found in the genomic data. (*B*) The percentage of GFZF peaks that overlap with HMR peaks and the percentage of HMR peaks that overlap with GFZF peaks increase with HMR overexpression. (*C*) Heatmap for HMR enrichment at GFZF peaks under the three conditions, sorted by HMR signal strength in the induced condition. Each line represents the same 4 kb stretch around a GFZF peak across the three conditions. The GFZF heatmap shows the GFZF signal strength at each of these positions. (*D*) Histogram of relative enrichment of HMR around GFZF peaks at all GFZF peaks for the three conditions.

To test these ideas, we overexpressed HMR and LHR in *D. melanogaster* S2 cell culture, and performed ChIP-Seq with HMR to identify its genomic binding sites and compared them to endogenous binding sites. We then compared these data with GFZF ChIP-Seq profiles from a previously published study (Baumann et al. 2018). When both proteins are overexpressed, HMR localizes to new sites throughout the genome in a pattern consistent with our observations of HMR localization to GFZF binding sites in hybrids. This pattern can be seen in representative examples (fig. 5*A*, supplementary figs. S8–S10, Supplementary Material online) and by examining the global levels of HMR and GFZF overlap (fig. 5*B*). GFZF has been previously described as a transcriptional activator, and therefore it is possible that the pattern of increased HMR association with GFZF sites is due to a preference of HMR for open chromatin. To address this, we compared HMR binding sites to the location of ATAC sites, which represent accessible chromatin (Albig et al.) (supplementary fig. S11, Supplementary Material online). We find that there is no significant change in HMR association with ATAC sites in any condition, indicating that its increased association with GFZF is not driven by a simple preference for open chromatin. By examining all the sites that are classified as GFZF and HMR overlapping sites when HMR expression is induced, it is clear that HMR is enriched at GFZF sites in the induced condition (fig. 5*C* and *D*). Though the levels of HMR expression in these S2 cells is likely greater than the levels in hybrids, these data show that expressing surplus HMR is sufficient to induce HMR mis-localization to GFZF binding sites.

## Discussion

*Hmr*, *Lhr*, and *gfzf* genetically interact in a single hybrid male lethal incompatibility between *D. melanogaster* and *D. simulans* (Barbash et al. 2003; Brideau et al. 2006; Phadnis et al. 2015). All three genes evolve rapidly under recurrent positive selection and encode chromatin binding proteins. Although HMR and LHR physically bind each other and function together in a single complex, the connection between either of these proteins to *gfzf* remains mysterious. Our results indicate that GFZF localizes to discrete genomic locations, but the binding profiles of GFZF do not overlap with those of HMR. These proteins come close to each other at chromosome ends, where GFZF is heavily enriched at the retrotransposon arrays that form fly telomeres and HMR/LHR function together in the telomere capping complex. Despite this close proximity of GFZF with HMR at chromosome ends, GFZF does not overlap with HMR and instead the two proteins reflect the boundary between the retrotransposon arrays at telomeres and the telomere capping complex. There is some evidence that HMR and LHR localize to the centromere or pericentric heterochromatin (Thomae et al. 2013; Blum et al. 2017). In our images, we do not find GFZF near these regions in polytenes, but this approach may not provide a complete picture when assaying the centromeric region of the genome as the chromocenter and pericentric heterochromatin are underreplicated in polytene chromosomes.

However, in interspecies hybrids these proteins meet each other in the form of altered chromatin localization of HMR to GFZF binding sites. This change is easily observed in the form of HMR localization to the ends of chromosomes where GFZF is prominent, but also at other euchromatic GFZF binding sites. Previously, HMR has been observed to localize to additional sites in hybrids compared with its within species binding patterns, but the nature of these sites remained unknown (Thomae et al. 2013). These additional sites can now at least partially be accounted for through GFZF binding sites. Our results show that reducing the expression of the incompatible *gfzf*^ *sim*^ allele restores the normal localization patterns of HMR. Although removing the *D. simulans* alleles of either *Lhr* or *gfzf* rescues hybrid male viability, removing *Lhr*^ *sim*^ does not restore the normal localization pattern of HMR indicating distinct roles for *Lhr* and *gfzf* in the hybrid incompatibility. Together, these results provide evidence for a new molecular interaction between the hybrid incompatibility factors involved in hybrid male lethality between *D. melanogaster* and *D. simulans*.

The observation that reducing *gfzf*^ *sim*^ restores normal localization of HMR raises the possibility that a novel physical interaction between GFZF ^sim^ and HMR may underlie the altered localization of HMR to GFZF binding sites in hybrids. However, our experiments with *D. melanogaster* cultured cells show that overexpressing *Hmr* and *Lhr* is sufficient to localize HMR to GFZF binding sites in the absence of *gfzf*^ *sim*^. A novel physical interaction between HMR and GFZF may, therefore, not be necessary for the altered localization patterns seen in hybrids. Instead, a natural affinity of surplus HMR for GFZF binding sites may be sufficient to explain the altered binding patterns observed in hybrids. *Hmr* and *Lhr* are known to be overexpressed in hybrids between *D. melanogaster* and *D. simulans* (Thomae et al. 2013). It is yet unclear how *gfzf*^ *sim*^ increases HMR binding to GFZF sites, whereas *gfzf*^ *mel*^ does not. It is possible that *gfzf*^ *sim*^ works with other chromatin modifying proteins and alters chromatin such that it is more accessible to HMR. Conversely, it is possible that *gfzf*^ *sim*^ directly or indirectly affects the expression of *Hmr* and/or *Lhr*, leading to their overexpression in hybrids.

The most obvious difference in GFZF localization between *D. melanogaster* and *D. simulans* using polytene analyses is at their telomeres. The *D. melanogaster* and *D. simulans* alleles of GFZF, however, are capable of binding to the telomeric retrotransposon arrays at telomeres from either species. From our data, it does not appear that the amino acid differences in GFZF between *D. melanogaster* and *D. simulans* contribute to their species-specific binding patterns. Instead, these results reflect the evolution of dramatic copy number differences in the telomeric retrotransposon arrays between the two species. Recent evidence suggests that the distribution of TAHRE retrotransposons may differ between *D. melanogaster* and *D. simulans*, though it is not yet known whether telomeres at specific chromosomes drive this signal (Saint-Leandre et al. 2018). Further experiments to explore the role of GFZF in telomere regulation may help determine how GFZF operates as a transcriptional coactivator, but also the nature of its localization to the highly repressed retrotransposon repeats of the telomere.

Although hybrids between *D. melanogaster* and *D. simulans* show increased expression of transposable elements including those at telomeres, this is not thought to be directly responsible for hybrid male lethality (Thomae et al. 2013; Satyaki et al. 2014). However, the need to control telomeric retrotransposons may have provided the substrate for the rapid evolution of *Hmr*, *Lhr*, and *gfzf*. Telomeric retrotransposons must replicate for cells to live, yet rampant replication may cause widescale genome instability. There is evidence that telomere length is highly variable within *D. melanogaster* (Wei et al. 2017), and that the telomere capping proteins have undergone significant divergence over the course of *Drosophila* evolution (Vedelek et al. 2015). Therefore, we speculate that the regulation of the number of retrotransposon repeats and the telomere/euchromatin boundary might be important stages of intragenomic conflict. All three hybrid incompatibly genes are positioned to be involved in both conflicts—in hybrids, the extension of HMR into the GFZF regions of telomeres could may reflect the loss of a boundary between the two regions. These ideas provide guidance for further experiments into understanding the functional consequences of the altered localization of HMR to GFZF binding sites, particularly at telomeres. Together, our results suggest that a deeper understanding of the molecular interactions of *gfzf* may shed light on telomere length regulation through retrotransposon copy number control, and its relation to the evolution and molecular mechanisms of hybrid incompatibilities. Our results reinforce the idea that dissecting the cellular processes that are disrupted in hybrids can provide clues into the molecular mechanisms and the evolutionary forces that drive the formation of hybrid incompatibilities between species.

## Materials and Methods

### Drosophila Husbandry and Strains

To produce hybrids between *D. melanogaster* and *D. simulans*, we set crosses of 3–4 virgin female *D. melanogaster* and 7–9 two-day old *D. simulans* males. We allowed mating to occur at 25 °C for 2–3 days, and then transferred the vials with progeny to 18 °C to develop. Unless stated otherwise, our standard *D. melanogaster* line was *yw*, and our standard *D. simulans* line was *w*^*501*^. For our polytene analyses, we set up all crosses with 3–4 males and females at 25 °C for 2 days and reared the progeny 18 °C until large third instar larvae were ready for dissection.

### Transgenic Flies

To construct flies that expressed EGFP.GFZF, we cloned the *gfzf* locus from both *D. melanogaster* and *D. simulans* into plasmid vectors for transformation into fly lines and created stable stocks. Briefly, we amplified 1KB upstream of the transcript start site of *gfzf* and 1KB downstream of the stop codon of *gfzf* in order to capture any local regulatory elements. We used Gibson Assembly (Gibson et al. 2009) to transform this segment of the *gfzf* locus with the sequence for EGFP (fused to the *N*-terminus of *gfzf* with a short linker sequence) into the pattB and pCasper4 vectors. Injections of these vectors into PBac{y[+]-attP-3B}VK00002 (Bloomington Stock 9723) by phiC31 transgenesis for *D. melanogaster* and *w^501^* by *P*-element mediated transgenesis for *D. simulans* were performed by The Best Gene Inc. We confirmed the successful integration of these vectors via an *mW* marker, and the expression of EGFP.GFZF by wide-field fluorescence microscopy. In all cases we found that the 1KB upstream and downstream regulatory regions were sufficient alone to drive expression of EGFP.GFZF at a moderate level in every cell type we examined (as compared with the native fluorescence of other transgenes we have used in our lab). All sequence information are available in the Supplementary Material online The UAS.*gfzf*^ *sim*^ RNAi strain was developed for a previous study (Phadnis et al. 2015), and uses a shRNA construct to target a 6 bp deletion that is a fixed difference between *D. melanogaster* and *D. simulans gfzf.*

### Polytene Chromosome Analysis

We isolated polytene chromosomes from the salivary glands of third instar larvae. We extracted the salivary glands into PBS and fixed them in 45% acetic acid and 4% paraformaldehyde for 30 s. We then softened the salivary tissue in 22.5% acetic acid and 50% lactic acid for 1 min. We flattened the glands and cleared tissue under a wide-field dissecting scope between a coverslip and a glass slide. We fixed the tissues to the slide by freezing the sample with liquid nitrogen and removing the coverslip using a swift flicking motion. We stored samples in PBS at 4 °C for no more than 3 h before proceeding to immunofluorescent antibody staining.

### Antibodies and Immunofluorescence

To fluorescently stain polytene chromosomes, we first washed them with PBS with 0.3% TritonX-100 (PBT). We then blocked them for 10 min with normal goat serum, and incubated them in with a primary antibody solution for 1 h and 20 min at room temperature. The rabbit anti-GFZF primary antibody was developed by Covance against the target RRDVAEPAKGAQPDC. For our primary antibody concentrations, we used the following: rabbit anti-GFZF 1:200, rat anti-HMR 1:20 (Thomae et al. 2013), mouse anti-HP1 (DSHB C1A9), and chicken anti-GFP 1:200 (Abcam 13970). We washed the samples six times with PBT before incubating them in a secondary antibody solution for 1 h and 20 min at room temperature. All secondary antibodies were generated from goats and used at a concentration of 1:1,000. We washed away the secondary antibody with three washes of PBT. To stain DNA, we incubated the samples with Hoechst 32258 (Thermo Fisher) at 1:1,000 in PBT for 4 min. We washed twice more with PBS before mounting the samples under glass coverslips in Fluoromount-G. We imaged all our samples using the Zeiss LSM880 Airyscan system, and processed all images using the Zen software from Zeiss and the Fiji package in ImageJ.

### Colocalization Analysis

To analyze the colocalization of HMR and GFZF in our polytene images, we measured the frequency of co-occurring bands. To do this, we drew a segmented line along DNA using tools in Fiji (a package manager for ImageJ), and generated a table of the intensities of the HMR and GFZF signal from their respective channels. To pick the location of our lines, we avoided regions that contained large, continuous stretches of signal from either channel, as to not artificially inflate the amount of colocalization. We also picked segments that contained at least one prominent band from both channels to ensure that signals could be clearly separated from background noise. We used the DNA channel to find continuous segments of DNA. For each condition, we measured at least five polytene preps from separate larvae. For each polytene, we made three measurements on different segments of the polytene and averaged the three measurements. To process the data, we wrote a script in python3 to detect local maxima (peaks in fluorescent intensity that represent bands) and count the co-occurrence of maxima in both samples versus the total number of maxima observed (supplementary fig. S7, Supplementary Material online). We avoided double-counting by setting a minimum distance between local maxima (0.6 μM), and set a background threshold as 20% of the maximum intensity value to avoid noise in the fluorescent signal. We tested for statistical difference in our samples by implementing a permutation test using python, with the test running for 100,000 permutations of the different data sets to test for a difference in the mean between each sample and our wild-type control.

### Cell Culture

*Drosophila* SL2 cells stably transfected with FLAG-HA-HMR and Myc-LHR under a CuSO_4_ inducible promoter (pMT) were grown at 26 °C in Schneider *Drosophila* medium (Invitrogen) supplemented with 10% fetal calf serum and antibiotics (100 units/ml penicillin and 100 μg/ml streptomycin). Transfected cells were selected with 20 μg/ml Hygromycin B. Cells were grown to confluence in 550 ml flasks and induced for 20–24 h with 250 μM CuSO_4_ before harvesting for chromatin immunoprecipitation.

### Immunoprecipitation

Nuclear extracts from 0 to 12 h Oregon R embryos were subjected to immunoprecipitation as follows. α-FLAG immunoprecipitation was performed using 20 μl of packed agarose-conjugated mouse α-FLAG antibody (ANTI-FLAG M2 Affinity gel, A2220 Sigma-Aldrich). α-GFZF IP was performed using 12 μl of rabbit antibody noncovalently coupled to 30 μl of Protein A/G Sepharose. Unspecific rat IgG noncovalently coupled to 30 μl of Protein A/G Sepharose through a bridging rabbit antirat IgG (Dianova, 312-005-046) and Protein A/G Sepharose alone (beads-only) were used as mock controls, in mass-spectrometry and immunoblots experiments, respectively. The steps that follow were the same for all the immunoprecipitations and were all performed at 4 °C. The antibodies coupled with the solid phase were washed three times with IP buffer (25 mM Hepes pH 7.6, 150 mM NaCl, 12.5 mM MgCl_2_, 10% Glycerol, 0.5 mM EGTA) prior to immunoprecipitation. Nuclear extracts were treated with benzonase (MERCK 1.01654.0001) and rotated 1 h end-over-end at 4 °C to digest nucleic acids. To remove insoluble material the digested extracts were centrifuged for 10 minutes at 20,000 × g and the supernatant was transferred to new tubes containing the antibody-beads solution (IP buffer complemented with a cocktail of inhibitors containing Aprotinin, Pepstatin, Leupeptin, 0.25 μg/ml MG132, 0.2 mM PMSF, 1 mM DTT was added up to a total volume of 500 μl) and end-over-end rotated for 2 h (α-FLAG) or 4 h (α-GFZF and IgG). After incubation the beads were centrifuged at 400 × g and washed three times in IP buffer complemented with inhibitors and three additional times with NH_4_HCO_3_ before in beads digestion.

### ChIP-Seq

Chromatin immunoprecipitation was essentially performed as in previous work with HMR (Gerland et al. 2017). For each ChIP reaction, chromatin isolated from 1 to 2 × 10^6^ cells was incubated with rat anti-HMR 2C10 antibody precoupled to Protein A/G Sepharose through a rabbit IgG antirat. The samples were single-end, 50 bp sequenced with the Illumina HiSeq2000. An overview of all ChIP-Seq samples used, a list of HMR peaks used for further analyses is as well as all sequencing data are publicly available as described below. The raw reads were aligned to the *D. melanogaster* genome assembly (UCSC dm6) using Bowtie2 (2.2.9) and filtered for uniquely mapped reads using samtools (1.3.1) (Li et al. 2009). Input and sequencing depth normalized tracks were generated by Homer (4.9) and visualized using R graphics. Peak calling was performed using HOMER 4.9 with parameters –style factor –F 2 –size 200 for HMR and –style factor –F 6 –L 6 –size 200 for GFZF. ChIP-Seq profiles (bedgraph) and peaks (bed) were imported to R using the rtracklayer package (version 1.38.3). Profiles were converted to coverage vectors using the coverage function from the GenomicRanges package (version 1.30.3) and example regions were plotted as polygons (base graphics). Peak centered matrices were generated from coverage vectors using the coverageWindowsCenteredStranded function from the tsTools package (version 0.1.0, https://github.com/musikutiv/tsTools; Last accessed on May 2016). The matrices were log2-transformed and visualized as heatmaps using the image function or as composite plots by plotting the mean across peaks for each position using base graphics. Venn diagrams of peak overlaps were created using the Vennerable package (version 3.1.0.9). The endogenous HMR binding data set (GSE86106, Gerland et al. 2017) and the GFZF binding data set (GSE105009, Baumann et al. 2018) are derived from NCBI GEO.

### Data Access

All proteomics data are available in the Project PXD010712 in the PRIDE archive of EMBL-EBI. All HMR ChIP-sequencing data are available in the Project GSE118291 in the GEO archive of NCBI.

## Supplementary Material

Supplementary data are available at *Molecular Biology and Evolution* online.

## Supplementary Material

msz105_Supplementary_Data
