# Supplementary material for: Altered Localization of Hybrid Incompatibility Proteins in Drosophila
Source: Mol Biol Evol. 2019 Apr 30;36(8):1783–92. doi: 10.1093/molbev/msz105 (PMC6657725; doi:10.1093/molbev/msz105)
Supplement: msz105_Supplementary_Data [file msz105_supplementary_data.pdf]

Supporting Information for

Altered localization of hybrid incompatibility proteins in *Drosophila*

Cooper, J.C. [1], Lukacs, A. [2], Reich, S. [1], Schauer, T. [2], Imhof, A. [2, 3], Phadnis, N [1]*.

[1] School of Biological Sciences, University of Utah, Salt Lake City, UT 84112, USA.

[2] Institute for Molecular Biology, Biomedical Center (BMC), Faculty of Medicine, LMU Munich, Germany.

[3] Center for Integrated Protein Science Munich (CIPSM), Ludwig-Maximilians-Universität Müchen, Munich, Germany.

*Corresponding author

E-mail: nitin.phadnis@utah.edu

Supplemental Figure 1

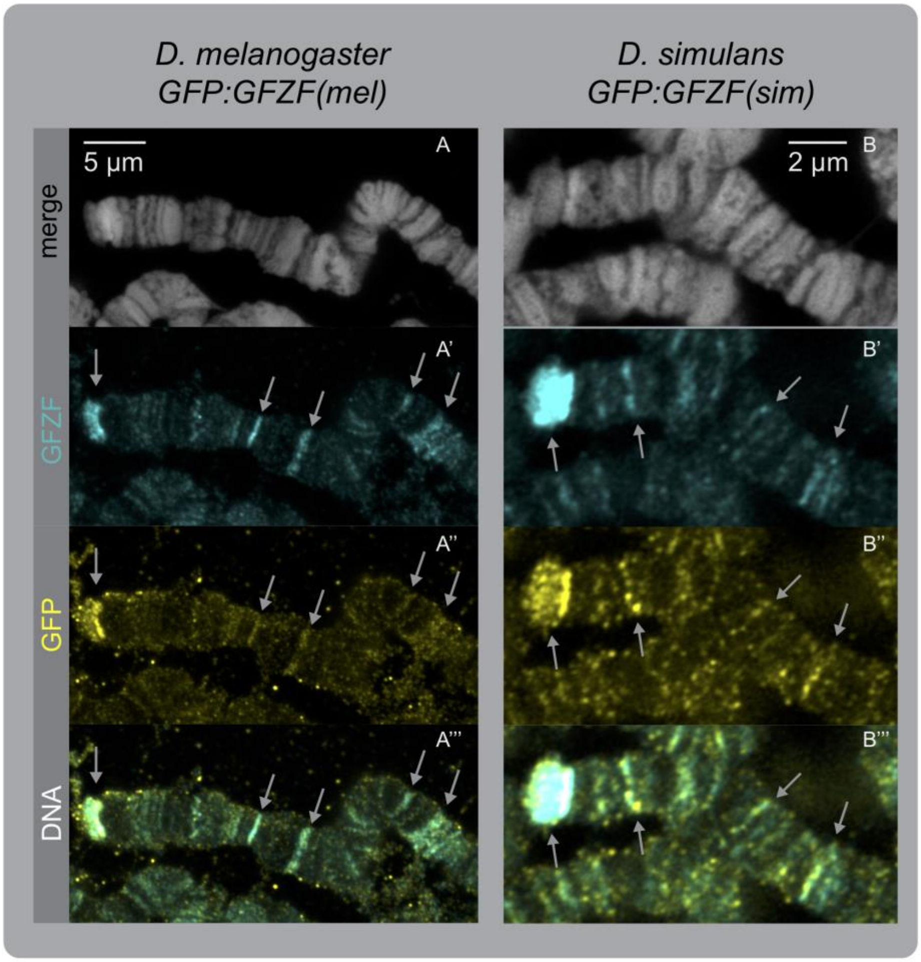

Supplementary figure S1. GFZF antibody binds GFZF^{mel} and GFZF^{sim}

Polytene chromosomes from transgenic lines with EGFP.GFZF^{mel} and EGFP.GFZF^{sim} co-stained with anti-GFP and anti-GFZF antibodies. White arrows point to strong bands of co-localization. Chromosomes were chosen due to species specific strong patterns of GFZF localization for best overlap comparison. (A) X chromosome of *D. melanogaster* (B) Chromosome 2L of *D. simulans*

Supplemental Figure 2

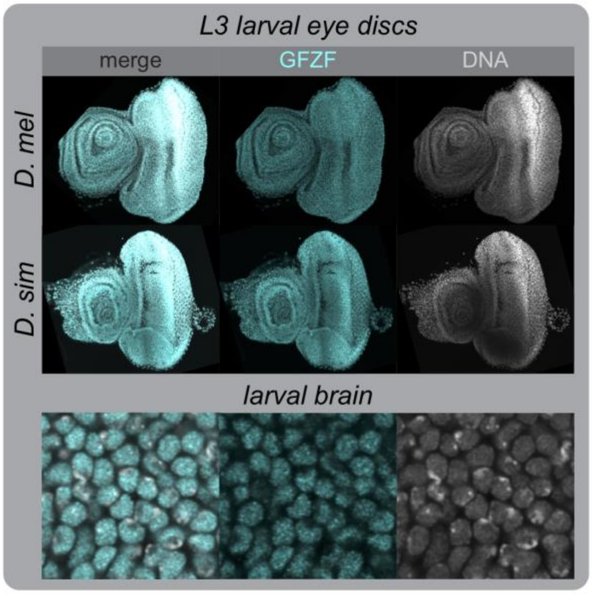

Supplementary figure S2. GFZF is expressed in the nucleus during development in *D. melanogaster* and *D. simulans*

Larval eye discs stained with anti-GFZF and Hoechst from *D. melanogaster* and *D. simulans*. Mid panels are zoomed in, single Z slices from the eye disc samples. In all these samples, GFZF co-localizes with Hoechst.

Supplemental Figure 3

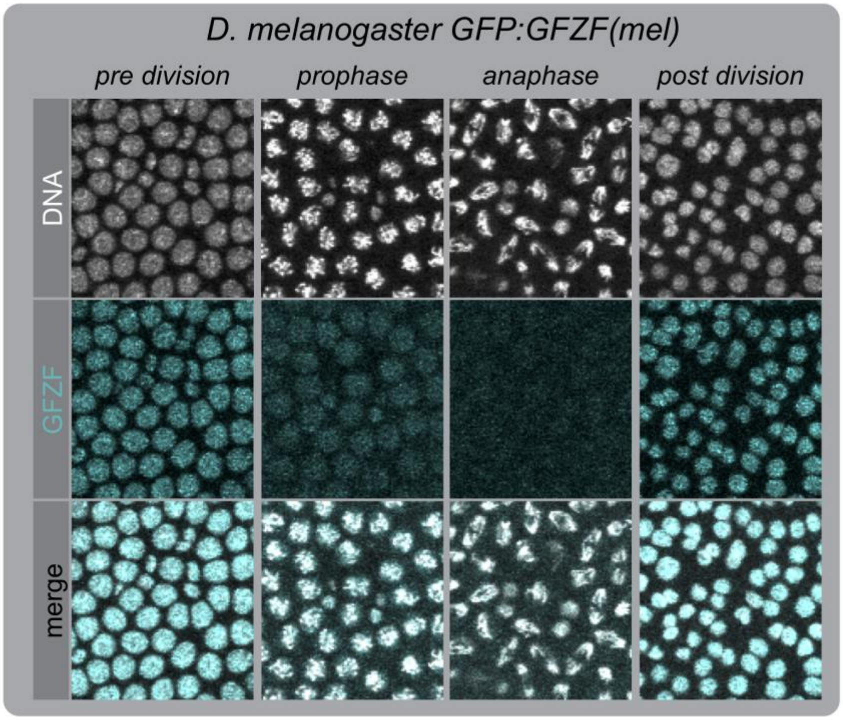

Supplementary figure S3. GFZF is absent from the chromatin of dividing cells

Live embryo imaging of EGPF.GFZF; H2av.RFP in *D. melanogaster* over the stage 6 – stage 7 cell division. Images were taken at 20 second intervals, and the panels here are representative of the cell cycle stages indicated in the legend. During mitosis, the EGFP signal depletes from the nucleus as chromatin condenses and is almost absent at the onset of anaphase. The signal returns early in the next prophase, and the process repeats.

Supplemental Figure 4

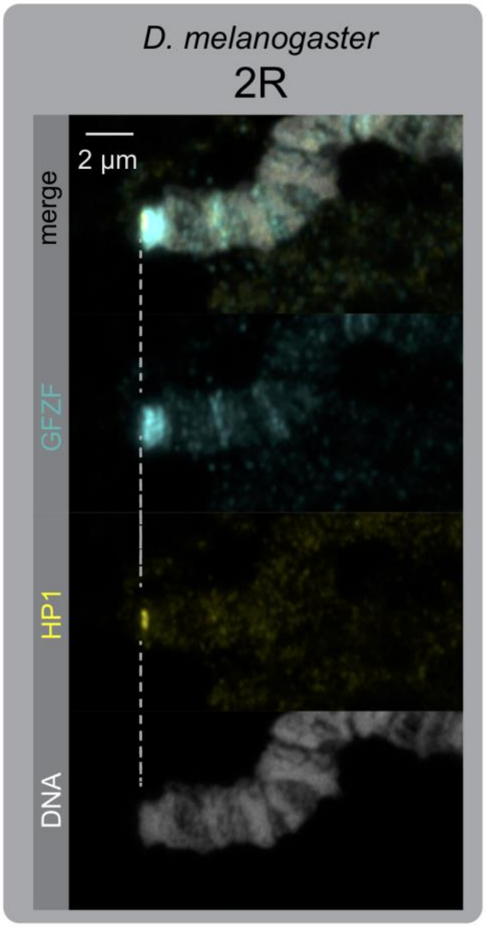

Supplementary figure S4. GFZF and HP1a do not co-localize at the ends of chromosomes, indicate GFZF does not bind the telomere cap

Co-staining for anti-GFZF and anti-HP1a. The alignment via the dashed line shows that the HP1a signal is just distal of the GFZF signal, similar to the pattern that we observe with GFZF and HMR.

Supplemental Figure 5

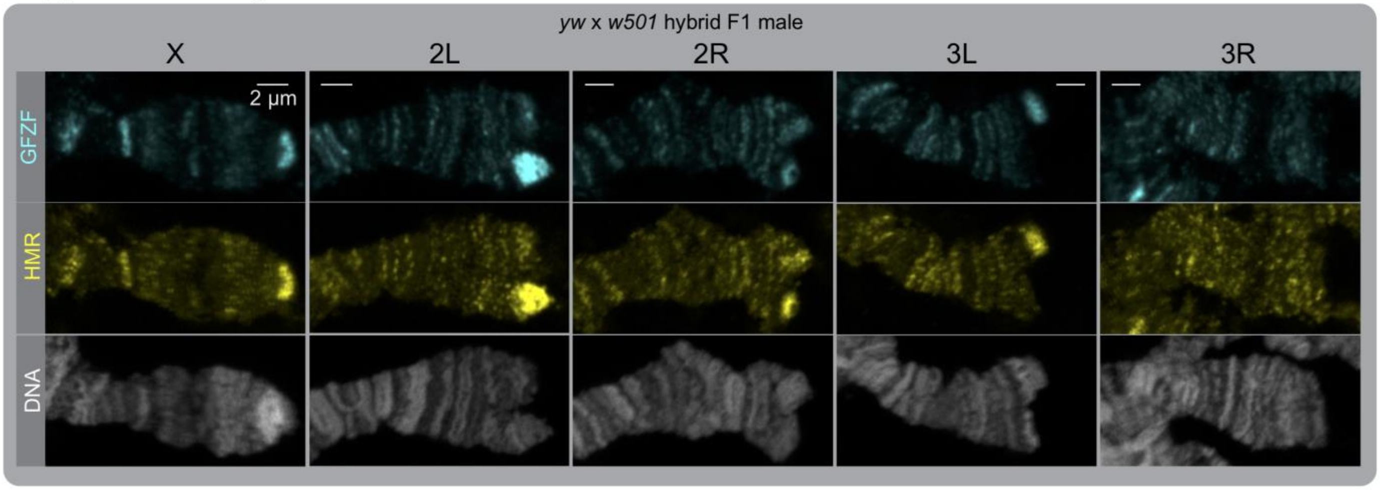

S5 Figure. GFZF localization at telomeres is different in hybrid F1 males

We analyzed polytene chromosomes from rare hybrid males that grow to be large 3rd instar larvae, and found that GFZF and HMR have staining patterns comparable to that of hybrid F1 females. Though these males grow as larvae, they do not produce viable adults. We confirmed that these are true hybrid males by two methods; the *yw D. melanogaster* X chromosome causes larval mouth hooks to be lighter in color, and the polytene karyotype contained only 1 X chromosome that stained for GFZF at the telomere, indicating that it is in fact the *D. melanogaster* X.

Supplemental Figure 6

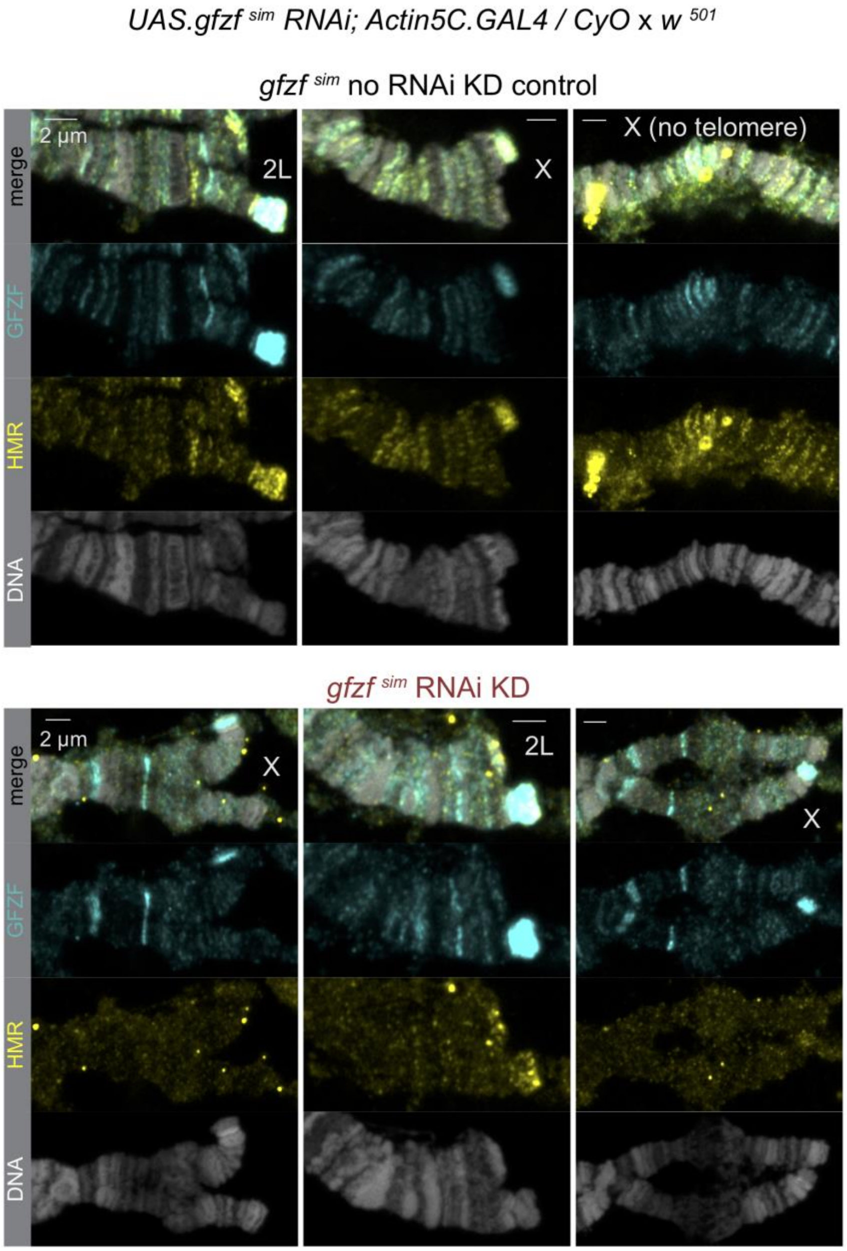

Supplementary figure S6. Additional images of *gfzf* ^{sim} knockdown hybrids

The top panels hybrid polytenes from control GAL4- samples. The bottom panels are hybrid polytenes are from GAL4+ *gfzf* ^{sim} knockdown samples. HMR staining and HMR / GFZF co-localization is reduced in the *gfzf* ^{sim} knockdown samples. Chromosome identities are given for each panel.

Supplemental Figure 7

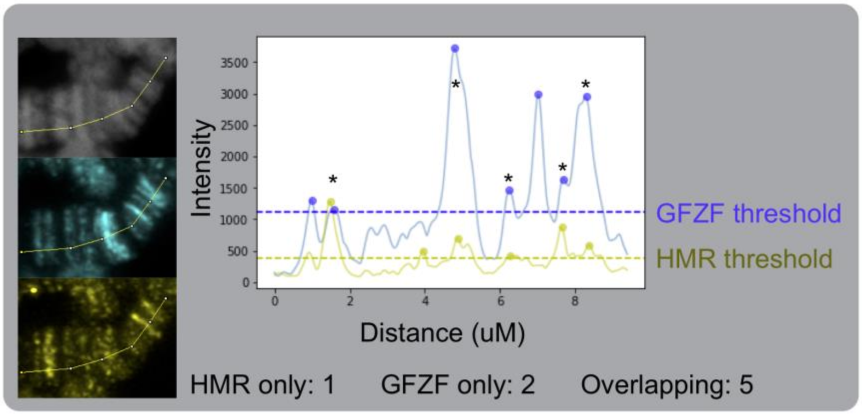

Supplementary figure S7. Example of GFZF / HMR overlap calculation process

Polytene fluorescent images analyzed for co-localization between GFZF and HMR along the DNA axis. The sample shown here is only several microns for sake if clarity – for our data presented in figure 4, we mapped ~150 microns of each polytene. (A) We used the segmented line tool in Fiji to trace the DNA line of the polytene chromosomes, and created fluorescent intensity outputs for the GFZF and HMR channels separately. (B) We determined local maxima for GFZF and HMR. (C) Tabulated the peaks of each channel individually and the peaks that were present in both channels. More details in methods.

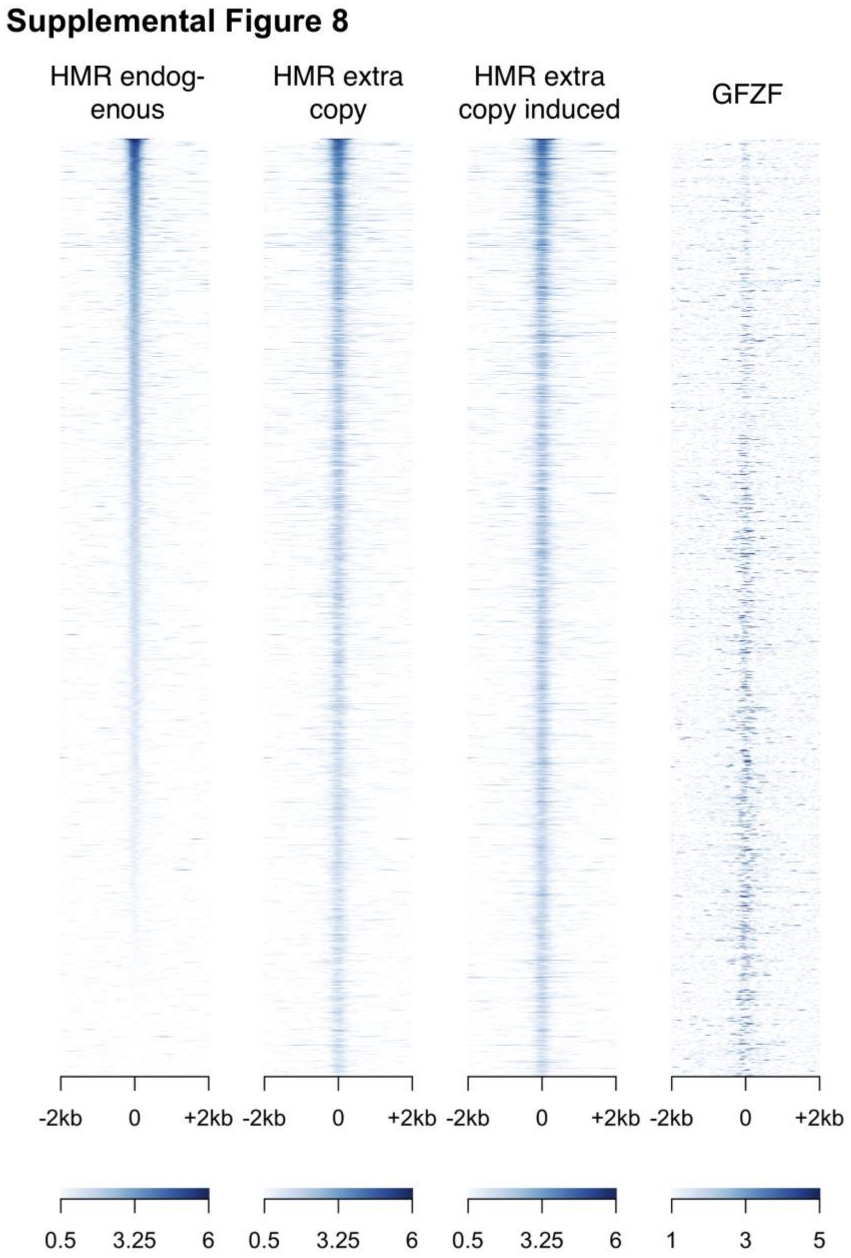

Supplementary figure S8. Heatmaps of HMR/GFZF signal all HMR bound sites in HMR over-expression experiments

The first three heat maps are for HMR signal. The final column represents the GFZF signal. All four columns are sorted by the strength of the HMR signal in the HMR over-expression induced state. When HMR is

over-expressed, new sites appear near the end of the heatmap (as inferred by lack of HMR signal in the control).

Supplemental Figure 9

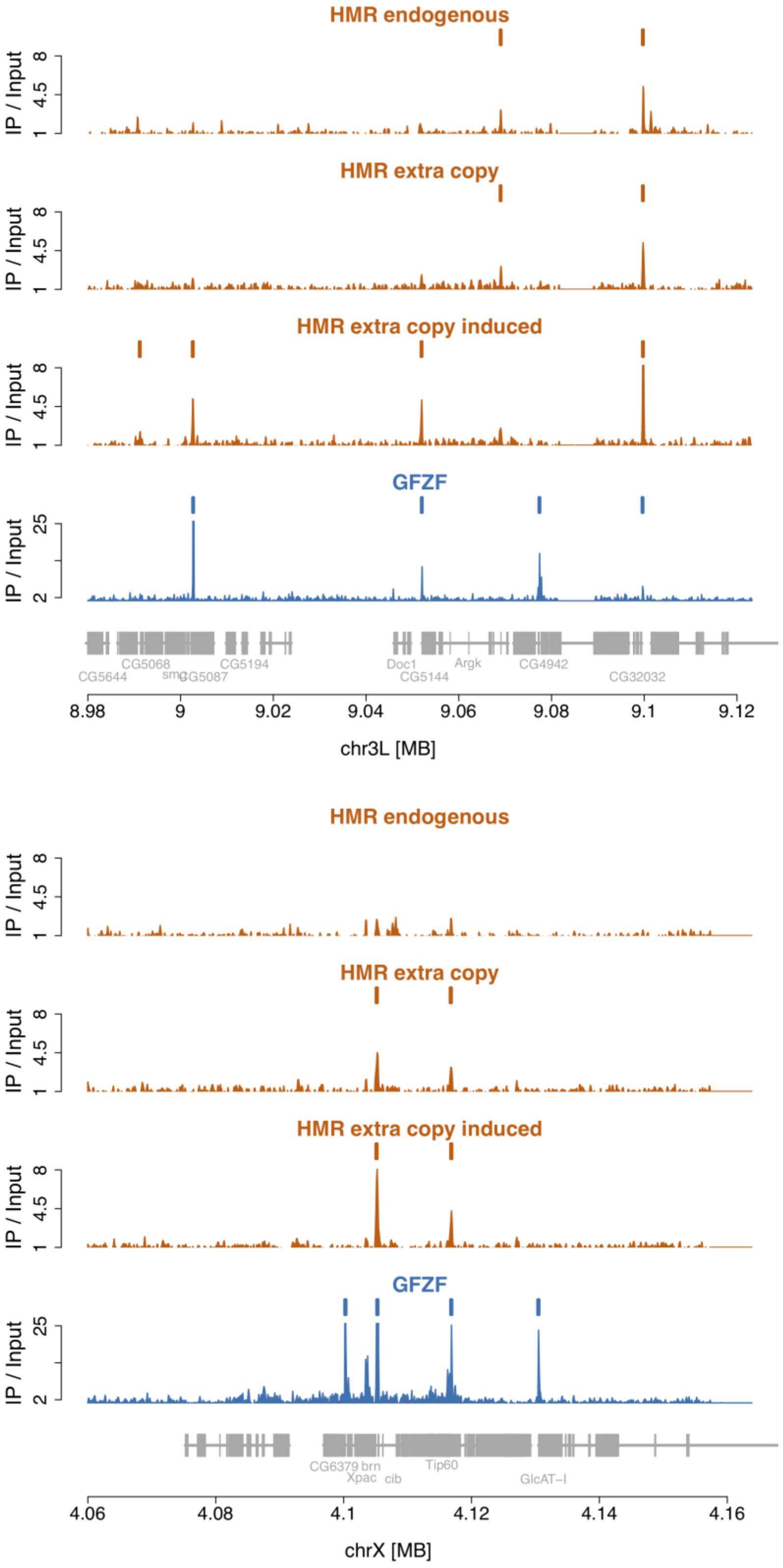

S9 Figure. Additional genomes browser windows for HMR over-expression

Additional windows to pair with Figure 5A. Peaks are marked with orange and blue bars for HMR and GFZF respectively.

Supplemental Figure 10

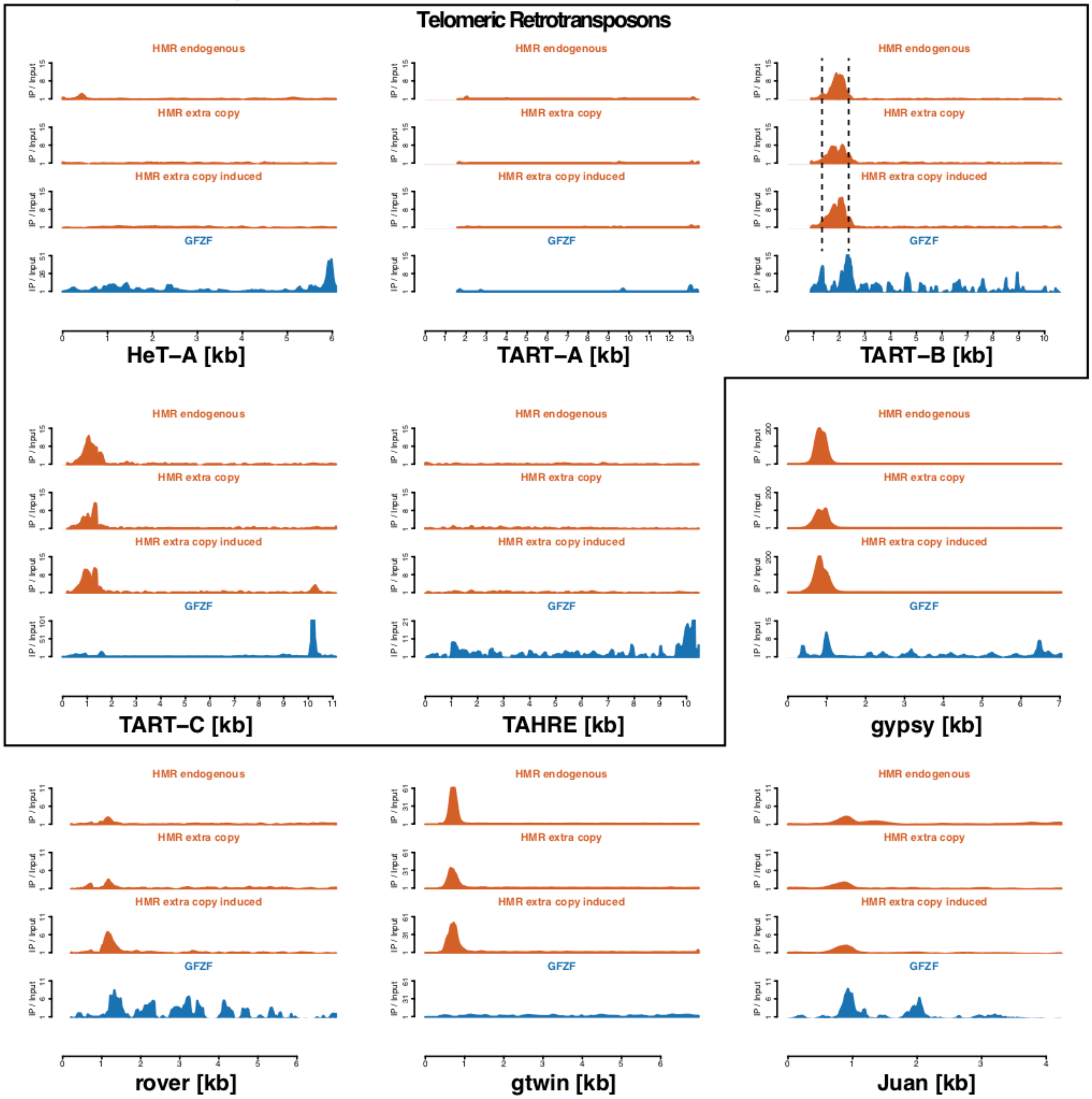

Supplementary figure S10. Genome browser windows for retrotransposons and other transposable elements.

Additional windows to pair with Figure 5A. Relative enrichment of the input samples for HMR native, additional copy uninduced, additional copy induced, and GFZF are shown for each telomeric TE and four

additional genomic TEs as a reference. Consistent with our polytene images, we found differential enrichment of GFZF and HMR in telomeric retrotransposon sequences, we find that HeT-A and TAHRE sequences show strong enrichment for GFZF but little to none for HMR, even with HMR over-expression (S10). We observe binding of GFZF and HMR in the TART-B and TART-C retrotransposons, but it appears that even in these elements their peaks of enrichment are largely non-overlapping.

Supplemental Figure 11

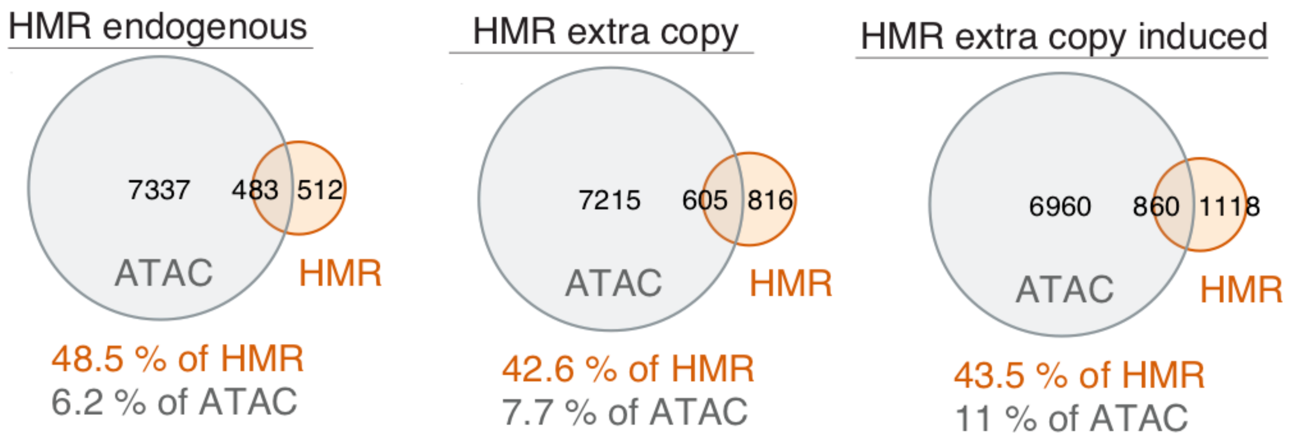

Supplementary figure S11. Overlap of HMR and ATAC peaks.

The number of peaks identified in ATAC-seq and HMR ChIP-seq from cell lines with endogenous HMR, 1 extra copy of HMR (uninduced), and the induced extra copy of HMR. The percentage of HMR peaks in ATAC peaks does not change significantly between the three conditions, indicating that an increase in HMR expression does not generally guide it to more open chromatin.
